# Correspondence—Stannous Gold

**Published:** 1882-03

**Authors:** Henry S. Chase


*Correspondence.*

St. Louis, Mo., February 13th, 1882.

Friend Hodgkin.—I was absent from home during December and part of January, and did not see the December Journal until to-day.

I am sure that you did not intend to do me injustice in your "Newest of Our Creeds." But on page 352 you say "Dr. H. S. Chase, working as a co-worker with Dr. Flagg, puts out an alloy simply of tin and gold and commends it as 'the best.'"

Now I say that I never said in any place whatever that my "Stannous Gold" was *the best.* I merely said that it was better as a *tooth saver* than all gold. Moreover I never sold an alloy of "gold and tin" merely. There was in "Stannous Gold" about forty parts each of tin and silver, and twenty parts of gold, making a twenty-five per cent. alloy.

Furthermore the formula for "Stannous Gold" was published by me in several dental journals. And I have never put on sale an alloy the formula for which I had not made public in the journals.

Very respectfully, your friend,

HENRY S. CHASE.

---

As to the above statement of Dr. Chase, no one would be further from knowingly misrepresenting him than the writer. He does not, however, recall ever seeing the formula for "Stannous Gold," and was clearly under the impression that it was, as its metallurgic title suggests, tin and gold. It should certainly not carry such an equivocal ensign. Call it by its full name, Doctor. H.

